# CTA-guided preoperative perforator selection for knee soft tissue reconstruction

**DOI:** 10.1186/s12893-026-03966-1

**Published:** 2026-06-18

**Authors:** Xuebiao Yang, Te Cai, Xingcheng Dai, Min Zhou, Xinyu Tian, Yan Shi, Yuexian Xu, Shen Xia, Xiaoqing He

**Affiliations:** 1 Kunming Medical University, Kunming, 650500 China; 2 Department of Orthopedic Surgery, 920th Hospital of Joint Logistic Support Force of Chinese PLA, Kunming, 650032 China; 3 Dali Clinical Medical College, Dali, 671000 China

**Keywords:** Computed tomography angiography (CTA), Perforator flap, Pedicled flap, Knee reconstruction, Soft tissue defect

## Abstract

**Background:**

Perforator flaps represent an effective and versatile option for the reconstruction of soft tissue defects around the knee. However, interindividual variability in perforator origin, course, and pedicle characteristics often complicates flap selection and increases intraoperative uncertainty. This study aimed to evaluate the role of computed tomography angiography (CTA) in guiding perforator selection and supporting individualized preoperative planning for knee soft tissue reconstruction.

**Methods:**

Between August 2014 and March 2024, we retrospectively evaluated 16 patients who underwent 17 flap procedures after preoperative CTA for reconstruction of soft tissue defects around the knee. The location, course, and length of candidate perforators were evaluated using the Picture Archiving and Communication System (PACS). Based on comparative preoperative assessment, the most suitable perforator was selected for flap design.

**Results:**

Seventeen flaps were harvested in 16 patients, including 16 CTA-selected perforator-based flaps and one random-pattern local flap selected after CTA showed no suitable perforator. All 16 CTA-selected target perforators were identified intraoperatively as anticipated, yielding a target-perforator identification discrepancy rate of 0/16. Complete skin-paddle survival was achieved in 15 of 16 CTA-selected perforator-based flaps and in 16 of 17 flaps overall. One CTA-selected perforator flap developed partial distal necrosis and healed conservatively; no total flap loss occurred. The random-pattern local flap survived completely without complications.

**Conclusion:**

CTA provided a practical decision-support method for preoperative assessment of perforator anatomy around the knee. In this proof-of-concept retrospective Level IV case series, the structured CTA-guided workflow was feasible and showed qualitative concordance with intraoperative findings, supporting individualized flap planning according to defect characteristics and perforator anatomy. Because no Doppler-only, non-CTA, or historical control group was included, these findings demonstrate feasibility and anatomical concordance rather than measurable clinical superiority over other mapping modalities.

## Introduction

Soft tissue defects around the knee remain challenging to reconstruct because durable coverage must be achieved over a highly mobile joint while preserving function and minimizing donor-site morbidity [1]. Over recent decades, perforator-based and regional flap techniques have expanded reconstructive options for peri-knee defects, including anterolateral thigh (ALT)–based flaps, descending genicular artery–based flaps, genicular artery perforator flaps, saphenous artery perforator flaps, and sural system–related perforator flaps [2–6]. Nevertheless, the breadth of available options also reflects the absence of a universally optimal flap across diverse defect patterns, local tissue conditions, and patient-specific vascular anatomy. We propose a preoperative, perforator-centered decision-support approach that operationalizes CTA findings into actionable selection criteria and flap-design choices for individualized peri-knee reconstruction.

A key reason for this lack of standardization is the substantial interindividual variability of perforator anatomy around the knee [7]. Anatomical mapping studies of the anterior knee region have demonstrated marked variability in perforator angiosomes and their supplying vessels, underscoring that perforator availability, caliber, and distribution cannot be assumed to be uniform across patients [8–10]. In clinical practice, such variability directly influences whether a planned perforator provides sufficient pedicle length, favorable orientation, and reliable perfusion to support safe flap rotation or advancement [11–13].

This uncertainty is clinically meaningful because unexpected perforator anatomy is a recognized driver of intraoperative strategy modification. In lower-extremity reconstruction, Lu et al. reported that planned ALT flaps could not be used in a subset of cases due to absent, small, or injured perforators, necessitating algorithm-based intraoperative contingency planning [14]. Similarly, in knee reconstruction with distally based ALT flaps, Du et al. described frequent intraoperative conversions in response to unexpected vascular findings, highlighting the practical limitations of decision-making that relies heavily on intraoperative exploration [15, 16]. Taken together, published reports suggest that improving preoperative certainty in perforator selection may help reduce the likelihood of unplanned conversions, additional dissection, prolonged operative time, and tissue trauma [17, 18]. However, these potential benefits require direct evaluation in comparative studies.

Accordingly, objective preoperative perforator assessment is essential for individualized planning. Handheld Doppler ultrasonography is widely used but provides limited information on perforator origin, deep course, pedicle geometry, and the spatial relationship between candidate perforators and the defect [18, 19]. In contrast, CTA offers high-resolution, three-dimensional visualization of perforator anatomy together with surrounding soft tissue and osseous structures, enabling quantitative preoperative comparison of candidate perforators [18, 20, 21]. In a prospective head-to-head comparison of lower-extremity perforator flap planning, Feng et al. evaluated CTA and color Doppler ultrasound against intraoperative verification, supporting the clinical utility of CTA for detailed perforator assessment [18].

Despite these advances, evidence remains limited regarding a practical CTA-guided strategy specifically tailored to peri-knee reconstruction that translates imaging into actionable selection criteria relevant to defect geometry and flap mechanics—such as proximity to the defect, pedicle length and caliber, anticipated rotation angle, and (when applicable) flow direction [17, 18]. Therefore, the present study incorporated CTA into preoperative planning for knee soft tissue defects to (i) systematically evaluate candidate perforators on PACS, (ii) apply a structured selection framework for flap design, and (iii) correlate CTA-based planning with intraoperative findings and postoperative outcomes.

## Methods

### Study design

This study was designed as a retrospective observational case series and represents Level IV evidence. Because no Doppler-only, non-CTA, or historical control group was included, the study was intended to evaluate feasibility, CTA–intraoperative concordance, and postoperative outcomes rather than to determine superiority over other perforator-mapping modalities. The reporting of this retrospective observational case series followed the Strengthening the Reporting of Observational Studies in Epidemiology (STROBE) guideline where applicable.

### Patients

Between August 2014 and March 2024, a total of 17 flap procedures were performed in 16 patients for reconstruction of soft tissue defects around the knee. The study cohort consisted of 12 male and 4 female patients, with a mean age of 45.9 years (range, 23–76 years). Seven patients presented with right-sided knee defects, and one patient had bilateral involvement. All patients provided written informed consent.

The etiologies of the soft tissue defects included traffic accidents (*n* = 4, including one bilateral case), postoperative infection following internal fixation (*n* = 3), post-burn ulcers (*n* = 3), machine-related injuries (*n* = 2), chronic ulcers (*n* = 2), and burns (*n* = 2). The anatomical distribution of the defects comprised anterior (*n* = 8, including one bilateral), medial (*n* = 4), anteromedial (*n* = 2), lateral (*n* = 2), and combined anterior and inferior knee regions (*n* = 1). Defect sizes ranged from 5 × 3 cm to 21 × 12 cm, and the interval between injury and reconstructive surgery ranged from 4 to 24 days (Table 1).

**Table 1:** Patient characteristics and flap procedures (16 patients, 17 flaps) treated with CTA-guided precise flap planning. DGF: Descending genicular artery perforator flap; RF: Random flap; SAF: Saphenous artery perforator flap; ALTF: Anterolateral thigh flap; SLGAF: Superior lateral genicular artery flap; PPF: Popliteal artery perforator flap; PAF: Peroneal artery perforator propeller flap; MSAP: Medial sural perforator propeller flap

Patient	Age	Sex	Cause of defect	Side	Time of injury to surgery (day)	Defect location	Defect size (cm)	Flap type	Flap size (cm)	Donor site closure	Follow-up time (months)	Flap outcome	Complications
1	23	Male	Postburn ulcer	Both	6	Anterior (both sides)	R6 × 4,L6 × 5	DGF	13 × 6(both)	Split-thinkness skin graft	10	Survival	None
2	76	Male	Chronic ulcer	Left	4	Anterior	8 × 4	RF	15 × 15	Direct closure	17	Survival	None
3	52	Male	Traffic accident	Left	9	Anterior medial	10 × 4	SAF	15 × 5	Split-thinkness skin graft	31	Survival	None
4	43	Male	Traffic accident	Left	13	Medial	15 × 11	ALTF	20 × 9	Direct closure	28	Survival	None
5	41	Female	Infection and necrosis after internal fixation	Left	5	Anterior	10 × 6	SLGAF	15 × 7	Direct closure	6	Survival	None
6	26	Female	Machinery accident	Left	8	Medial	12 × 7	PPF	18 × 7	Direct closure	14	Survival	None
7	70	Male	Postburn ulcer	Left	18	Lateral	7 × 4	PAF	25 × 4	Direct closure	6	Survival	None
8	49	Female	Postburn ulcer	Left	24	Lateral	5 × 3	MSAP	32 × 6	Split-thinkness skin graft	37	Survival	Hypertrophic scar and Scar ulcer
9	34	Male	Traffic accident	Left	10	Medial	6 × 4	SAF	12 × 6	Split-thinkness skin graft	16	Survival	None
10	66	Female	Infection and necrosis after internal fixation	Right	5	Anterior	9 × 6	ALTF	11 × 8.5	Direct closure	13	Survival	Bulky
11	54	Male	Infection and necrosis after internal fixation	Right	6	Inferior anterior	15 × 9	ALTF	17 × 9	Direct closure	44	Survival	None
12	48	Male	Burn	Right	7	Anterior	9 × 8	ALTF	14 × 9	Direct closure	7	Survival	None
13	39	Male	Machinery accident	Right	11	Anterior	11 × 9	ALTF	14 × 10	Direct closure	18	Survival	None
14	36	male	Traffic accident	Right	16	Anterior	7 × 5	ALTF	10 × 6	Direct closure	13	Survival	None
15	32	Male	Chronic ulcer	Right	7	Anterior medial	6 × 5	DGF	11 × 6	Direct closure	15	Survival	None
16	45	Male	Burn	Right	11	Medial	21 × 12	ALTF	21 × 10	Split-thinkness skin graft	10	Partial survival	Partial necrosis

All patients underwent preoperative CTA to evaluate potential donor and recipient sites, thereby supplementing conventional vessel localization methods based on clinical judgment [17, 18]. All procedures were conducted in accordance with the ethical principles of the Declaration of Helsinki. Written informed consent was obtained from all participants or, where applicable, from their legal guardians before enrollment.

### CTA acquisition

For patients included in this CTA-guided cohort, CTA was performed preoperatively to assess vascular anatomy relevant to knee soft tissue reconstruction. All patients were positioned supine with the lower extremities naturally extended. Iohexol (0.35 g/mL; 100 mL) was administered intravenously via the median cubital vein according to the institutional CTA protocol. Imaging was acquired using a 64-slice helical CT scanner with a slice thickness of 0.625 mm; tube voltage (120–140 kV) was adjusted according to patient habitus.

The scanning range extended from the abdominal aorta to the bilateral anterior tibial, posterior tibial, and peroneal arteries to evaluate the vascular axis and distal runoff, ensuring comprehensive visualization of donor- and recipient-site anatomy relevant to peri-knee reconstruction. CTA images were reviewed on the PACS for multiplanar reconstruction and three-dimensional assessment when needed.

### Surgical planning

Flap selection followed contemporary reconstructive principles, prioritizing patient safety, technical simplicity, like-with-like tissue replacement, surgical economy, donor-site morbidity, and recipient-site requirements [23, 24]. When more than one donor site was considered suitable, the technically simpler option with more compatible tissue characteristics was preferred.

### Evaluation of the surgical region

Preoperative CTA allowed integrated assessment of soft tissues, vascular structures, and osseous anatomy of the lower extremities. Using PACS, the defect size, shape, and location were documented, and the thickness of adjacent soft tissues and anticipated donor-site subcutaneous thickness were evaluated. The type and course of the main feeding vessels were assessed to support selection of an appropriate reconstructive option.

To standardize measurement of perforator location and estimated pedicle length, reproducible bony landmarks (e.g., the anterior superior iliac spine and fibular head) were used as reference points.

### Evaluation of perforators

Candidate perforators were evaluated on PACS by the operating reconstructive surgeon in consultation with a radiologist experienced in CTA interpretation. In this retrospective series, CTA interpretation was performed jointly for surgical planning rather than as an independent blinded multi-observer assessment; therefore, interobserver reliability was not analyzed. All identifiable perforators within the planned donor regions were initially mapped on PACS to minimize selection bias, and the number and source vessels of CTA-mapped candidate perforators were recorded for each patient (Table 2). For the selected perforator, the source vessel, location, course, estimated pedicle length, and vessel caliber were assessed on PACS when clearly measurable. Pedicle length was estimated along the visible course of the perforator from the source vessel to the fascial or skin-entry point using multiplanar reconstruction. Perforator caliber was assessed at the most clearly visualized segment near the source vessel or along the proximal perforator course. Candidate perforators were then screened using the following criteria: (1) clear visualization of the perforator and its source vessel on CTA, (2) an estimated pedicle length of ≥ 1 cm, and (3) the ability to design a flap of adequate dimensions to achieve complete defect coverage.

**Table 2:** CTA-mapped candidate perforators for each flap procedure (17 procedures in 16 patients)

Patient	Age	Sex	Side	Defect location	The numbers of the perforators	The source blood vessels of the perforators
1	23	Male	Both	Anterior (both sides)	R: 2 L: 1	R: 1 perforator: The descending genicular artery. 1 perforator: The descending branch of the lateral circumflex femoral artery. L: 1 perforator: The descending genicular artery.
2	76	Male	Left	Anterior	1	1 perforator: The transverse branch of the lateral circumflex femoral artery.
3	52	Male	Left	Anterior medial	1	1 perforator: The saphenous artery.
4	43	Male	Left	Medial	4	3 perforators: The descending branch of the lateral circumflex femoral artery. 1 perforator: The descending genicular artery.
5	41	Female	Left	Anterior	3	2 perforators: The lateral superior genicular artery. 1 perforator: The Popliteal Artery.
6	26	Female	Left	Medial	3	2 perforators: The popliteal artery. 1 perforator: The anterior tibial artery.
7	70	Male	Left	Lateral	2	2 perforators: The descending branch of the lateral circumflex femoral artery.
8	49	Female	Left	Lateral	2	1 perforator: The medial sural artery. 1 perforator: The peroneal artery.
9	34	Male	Left	Medial	1	1 perforator: The saphenous artery.
10	66	Female	Right	Anterior	4	2 perforators: The descending branch of the lateral circumflex femoral artery. 1 perforator: The transverse branch of the lateral circumflex femoral artery. 1 perforator: The popliteal artery.
11	54	Male	Right	Inferior anterior	3	2 perforators: The descending branch of the lateral circumflex femoral artery. 1 perforator: The transverse branch of the lateral circumflex femoral artery.
12	48	Male	Right	Anterior	2	2 perforators: The peroneal artery.
13	39	Male	Right	Anterior	3	2 perforators: The descending branch of the lateral circumflex femoral artery. 1 perforator: The transverse branch of the lateral circumflex femoral artery.
14	36	male	Right	Anterior	5	3 perforators: The descending branch of the lateral circumflex femoral artery. 1 perforator: The transverse branch of the lateral circumflex femoral artery. 1 perforator: The peroneal artery.
15	32	Male	Right	Anterior medial	2	1 perforator: The descending genicular artery. 1 perforator: The anterior tibial artery.
16	45	Male	Right	Medial	3	2 perforators: The descending branch of the lateral circumflex femoral artery. 1 perforator: The popliteal artery.

**Fig. 1:**
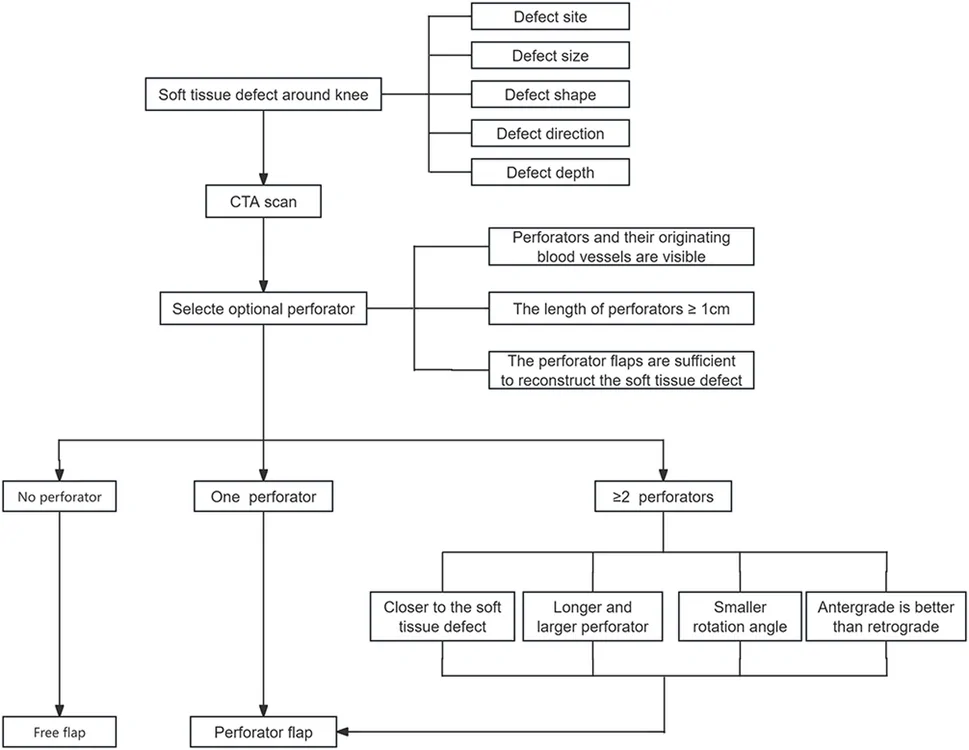
Flow chart of precise flap reconstruction of soft tissue defects around the knee guided by CTA

If only one perforator satisfied these criteria, it was selected for flap design. When two or more candidates were available, perforator selection was guided by practical considerations, including proximity to the defect, greater pedicle length and vessel caliber, a favorable pedicle orientation relative to the planned flap axis, and a smaller anticipated rotation angle to reduce the risk of kinking or torsion. The anticipated rotation angle was estimated preoperatively based on the planned pivot point and flap inset trajectory. If no suitable perforator was identified near the defect, an alternative reconstructive strategy, such as a random-pattern local flap or free flap reconstruction, was considered according to defect size, local tissue condition, and patient factors. Based on this systematic assessment, the optimal perforator was chosen for flap design (Fig. 1). These criteria were applied uniformly across all cases. Because exact rotation angles and complete quantitative measurements of all non-selected candidate perforators were not consistently recorded in this retrospective series, these variables were described qualitatively in the selection framework rather than added as inferred values.

### Flap design

Horizontal and vertical coordinates were established to localize the perforator penetration point at the donor site. Flap design was tailored to perforator location, with flap dimensions and orientation conforming to standard reconstructive principles. The positions of perforators and source vessels were marked on the skin surface. A pinch test was performed to assess the maximum feasible flap width, after which the preliminary outline of the flap was drawn.

### Flap harvest

Repeat debridement was performed to remove infected tissue, necrotic muscle, and devitalized bone. A paper template matching the defect was used to confirm that the preoperatively designed flap would provide adequate coverage.

Preoperative perforator mapping was used to guide an anterograde dissection strategy by facilitating early identification of the targeted perforator and planned pedicle course. This mapping was intended to support more focused dissection; however, operative efficiency and the extent of intraoperative exploration were not directly measured in this retrospective study. Flap elevation was performed according to the preoperative plan. One edge of the flap was initially incised to identify the perforator. For propeller flaps, perforators were dissected under magnification, ensuring a pedicle length of at least 1 cm. The source pedicle was dissected only to the extent required to achieve safe flap rotation or advancement and tension-free inset; routine skeletonization to the vascular origin was not performed unless required by the flap design. Additional perforators were ligated as needed while preserving the integrity of the flap vascular network. Cutaneous nerves were preserved whenever feasible to maintain flap sensibility. The flap was elevated beneath the deep fascia, and perfusion was assessed by dermal/capillary bleeding at the flap margin. After advancement or rotation, the flap was inset without tension. Donor sites were closed primarily whenever feasible; otherwise, split-thickness skin grafting was performed.

#### Operative time

The operative time for each procedure was not systematically recorded in this retrospective series. As a result, we are unable to report the median or range of operative time for the entire cohort. This limitation stems from the retrospective nature of the study, where certain intraoperative metrics were not consistently collected across all cases. Future prospective studies with standardized data collection protocols will be required to assess operative time as a comparative metric and to more thoroughly evaluate its relationship with other variables such as intraoperative exploration and decision-making.

### Intraoperative perfusion assessment

Intraoperative flap perfusion was assessed primarily by clinical parameters, including flap color, turgor, dermal bleeding, capillary refill, and absence of pedicle kinking or compression. Handheld Doppler, when used, served only as an adjunct to confirm pedicle or perforator flow and was not used as a comparative mapping modality or analyzed as a study endpoint.

### Postoperative management

Postoperatively, the affected limb was elevated to promote venous return. Standard postoperative care included thermal preservation, anti-infective therapy, and prophylactic anticoagulation. Knee immobilization was achieved using an external brace or plaster cast with the limb maintained in extension for 3–4 weeks to reduce the risk of wound dehiscence, joint exposure, and mechanical stress on the perforators.

Flap perfusion was closely monitored, and prompt intervention was undertaken in cases of vascular compromise. Patients were followed monthly for the first three months after surgery and subsequently by outpatient visits or telephone follow-up.

### Statistical analysis

Because this was a retrospective, single-center Level IV case series with a small and heterogeneous cohort and no Doppler-only, non-CTA, or historical control group, the analysis was descriptive. Categorical variables were summarized as counts and percentages. Continuous variables were summarized using the reliably available descriptive data. Age was reported as mean and range. Defect and flap dimensions were reported as length × width ranges, and follow-up duration was reported as a range. Median and standard deviation values were not calculated when complete patient-level source data required for reliable calculation were not consistently retrievable from the retrospective records. No imputation, extrapolation, or retrospective reconstruction of missing values was performed. Accordingly, no inferential statistical analysis was conducted.

## Results

**Fig. 2:**
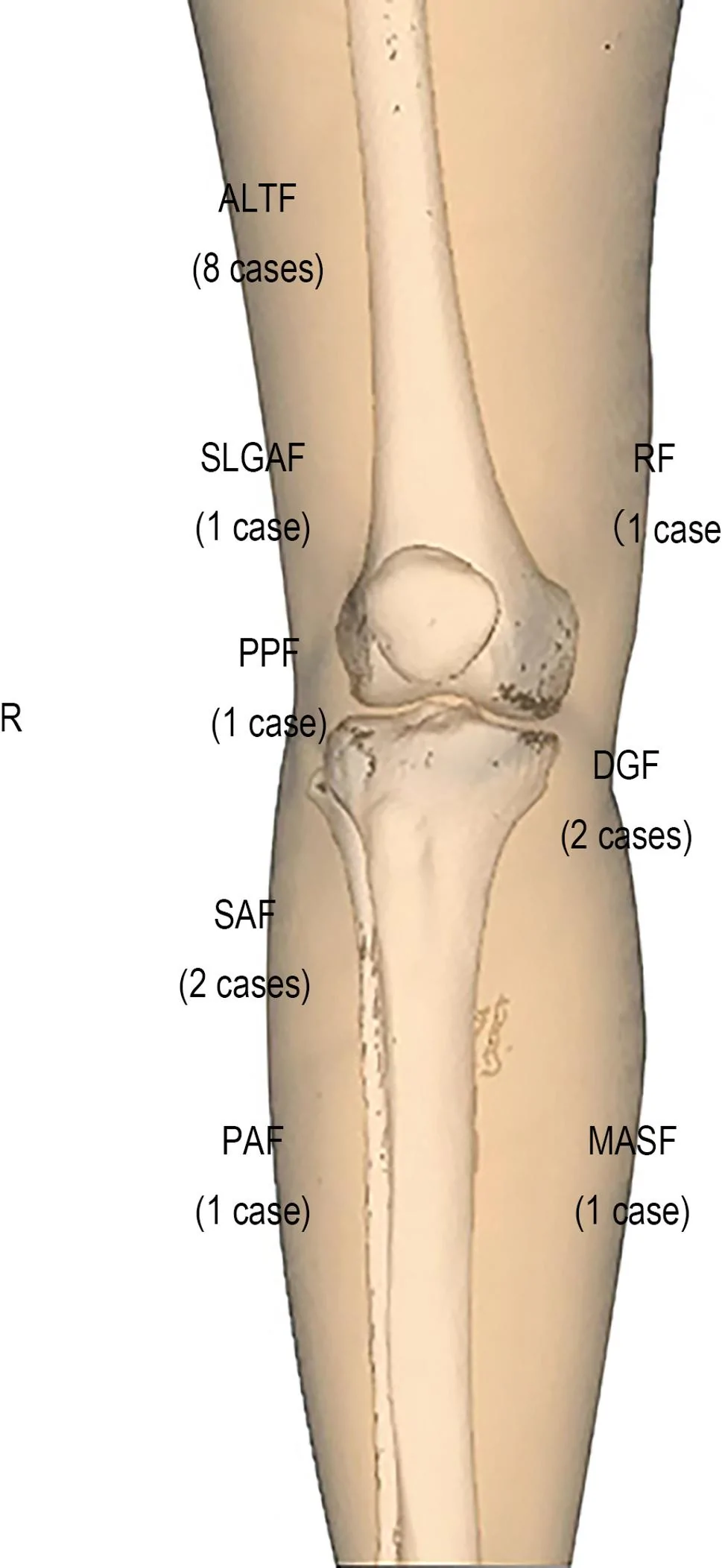
Front view of the right knee (distribution of position and quantity of each flap in this study)

A total of 17 flaps were harvested in 16 patients for reconstruction of soft tissue defects around the knee. The cohort included 12 male and 4 female patients. The mean patient age was 45.9 years, with an age range of 23–76 years. Preoperative defect dimensions, reported as length × width, ranged from 5 × 3 cm to 21 × 12 cm. Flap dimensions ranged from 10 × 6 cm to 21 × 10 cm. Follow-up duration ranged from 6 to 44 months. Among the 17 flaps, 16 were CTA-selected perforator-based flaps and one was a random-pattern local flap selected after CTA showed no suitable perforator for perforator-based flap design. The random-pattern flap was included to illustrate the role of CTA in preoperative reconstructive decision-making but was excluded from the primary analysis of CTA-selected perforator feasibility and CTA–intraoperative concordance. Eight defects were reconstructed using retrograde ALT flaps, two using descending genicular artery perforator flaps, and two using saphenous artery flaps. One case each was reconstructed using a superior lateral genicular artery perforator flap, a peri-popliteal perforator flap, a medial sural artery perforator propeller flap, and a peroneal artery perforator propeller flap. The anatomical distribution of the flaps around the knee is illustrated in Fig. 2.

Complete skin-paddle survival, defined as full flap viability without partial or total necrosis, was achieved in 16 of 17 flaps overall (94.1%). Among the 16 CTA-selected perforator-based flaps, complete skin-paddle survival was achieved in 15 flaps (93.8%). One CTA-selected perforator flap developed partial distal necrosis involving less than 30% of the flap and healed with conservative wound care, without debridement, grafting, or further surgical intervention. No total flap loss occurred. The random-pattern local flap survived completely without postoperative complications.

An uncomplicated postoperative course was observed in 14 of 17 flaps overall (82.4%) and in 13 of 16 CTA-selected perforator-based flaps (81.3%). The three minor postoperative complications all occurred in CTA-selected perforator-based flaps and included transient flap swelling in one case, donor-site scar ulceration in one case, and partial distal necrosis in one case. None of these complications required further surgical intervention.

By design, all selected target perforators met the predefined criterion of clear CTA visualization. Intraoperatively, all 16 CTA-selected perforators were identified as anticipated, and their location, course, and general anatomical characteristics were consistent with preoperative PACS findings, indicating qualitative CTA–intraoperative anatomical concordance in this series. The CTA–intraoperative discrepancy rate for target perforator identification was 0 of 16 CTA-guided perforator flaps (0%). No CTA-selected perforator was abandoned intraoperatively because of absence, inadequate caliber, pedicle injury, or insufficient perfusion. Intraoperative conversion to an alternative perforator or flap due to target-perforator infeasibility occurred in 0 of 16 CTA-guided perforator flaps (0%). Among the 16 CTA-selected perforator-based flaps, no intraoperative conversion or modification of the original flap design occurred. Because intraoperative perforator diameter and pedicle length were not measured using a standardized quantitative protocol in all cases, formal quantitative correlation analysis between CTA-estimated and intraoperative measurements was not performed.

At final follow-up (6–44 months), qualitative clinical follow-up and retrospective chart review did not document substantial functional loss or permanent functional limitation in any patient. Because knee motion and functional status were assessed clinically rather than using standardized range-of-motion measurements or validated functional scores, these findings should be interpreted as qualitative clinical observations rather than objective functional outcome measurements.

### Illustrative cases

The following cases illustrate how CTA informed preoperative perforator selection and flap design, with emphasis on imaging–intraoperative correlation, postoperative complications, and follow-up findings.

#### Case 1

**Fig. 3:**
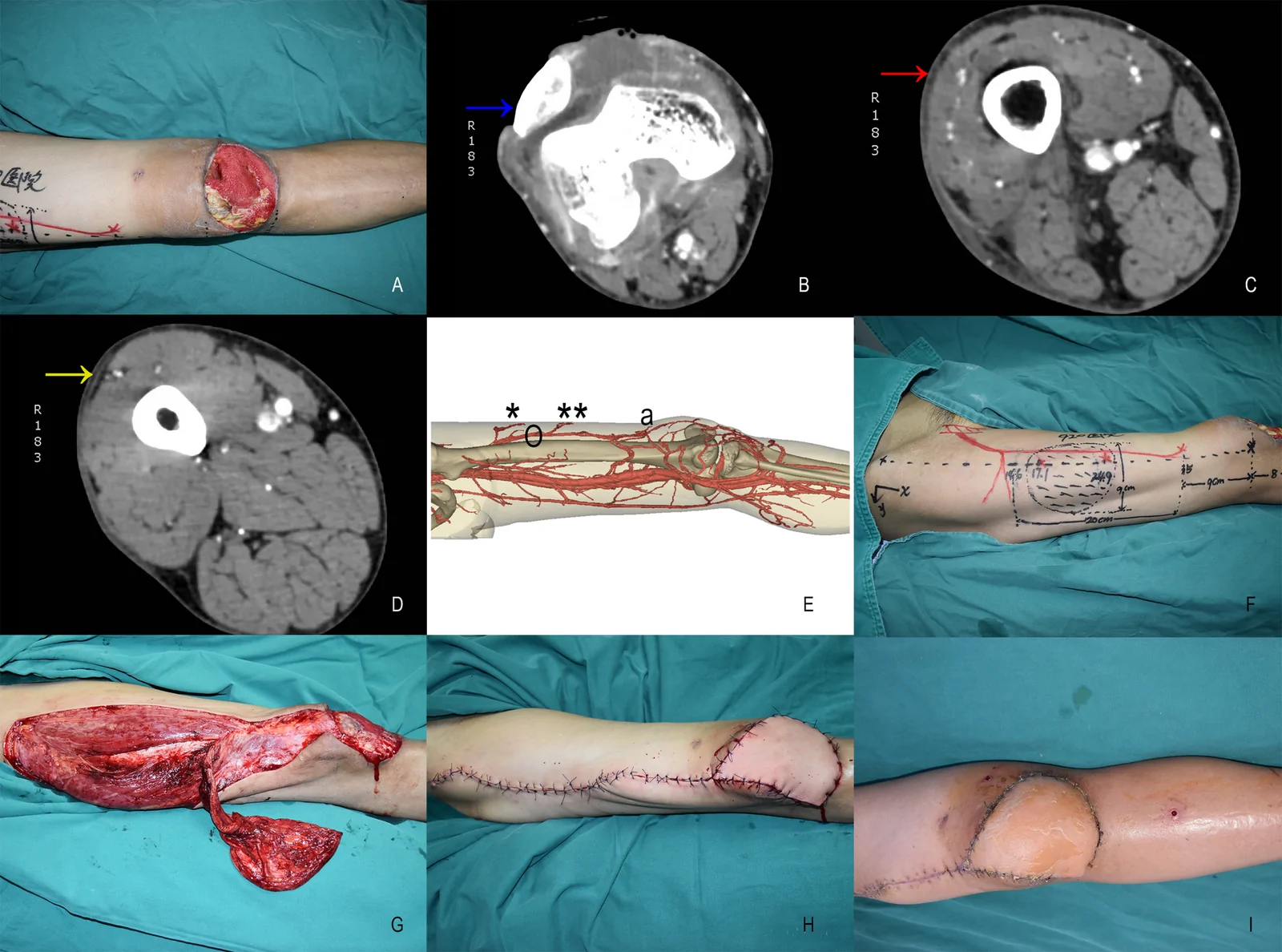
(**A**) Preoperative view showing a transverse soft tissue defect over the anterior aspect of the right knee. **B** Preoperative CTA image showing the location of the soft tissue defect, indicated by the blue arrow. The defect measured 9 × 8 cm, with patellar exposure. **C** Preoperative CTA image showing an anastomotic connection between the descending branch of the lateral circumflex femoral artery and the periarticular arterial network around the knee, indicated by the yellow arrow. **D** Preoperative CTA image showing the location of perforator 2, indicated by the red arrow. **E** Mimics 20.0 reconstruction demonstrating two perforators arising from the descending branch of the right lateral circumflex femoral artery. O, source vessel; *, perforator 1; **, perforator 2; a, anastomotic branch. **F** Design of a retrograde anterolateral thigh flap based on perforator 2. **G** Intraoperative view after harvest of a 14 × 9 cm retrograde anterolateral thigh flap without muscle dissection. **H** Immediate postoperative appearance after flap inset. **I** One-month postoperative photograph showing early satisfactory flap contour and texture

A 48-year-old male presented with a 9 × 8 cm anterior soft tissue defect on the right knee (Fig. 3A). Preoperative CTA (Fig. 3B) revealed two well-defined perforators arising from the descending branch of the lateral circumflex femoral artery. CTA also identified an anastomotic connection between this branch and the periarticular arterial network around the knee (Fig. 3C). Based on CTA data, perforator 2 was selected for flap design (Fig. 3D). Intraoperative findings were consistent with the CTA-based plan, and a 14 × 9 cm retrograde ALT flap was harvested (Fig. 3E and F). The flap was rotated to cover the defect, and the donor site was closed primarily without tension (Fig. 3G). The 1-month postoperative appearance is shown in Fig. 3I. At the final 7-month clinical follow-up, the flap remained stable, with satisfactory contour and appearance. Transient postoperative flap swelling occurred early after surgery and was managed conservatively with limb elevation and compression, resolving within two weeks.

#### Case 2

**Fig. 4:**
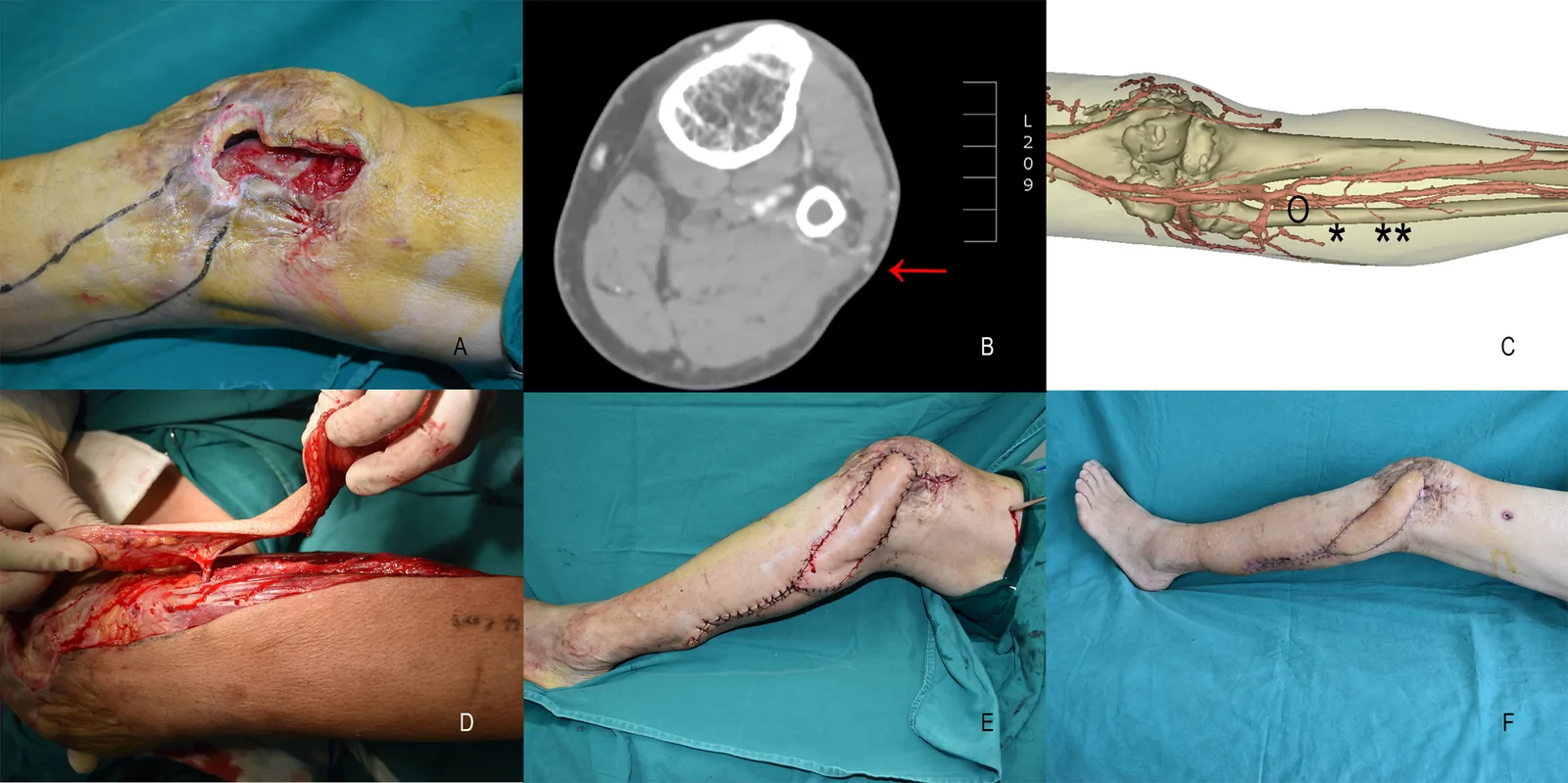
(**A**) Preoperative view showing a 7 × 4 cm soft tissue defect on the lateral aspect of the left knee. **B** Preoperative CTA image showing the location of perforator 1, indicated by the red arrow. **C** Mimics 20.0 reconstruction demonstrating two perforators arising from the peroneal artery. O, source vessel; *, perforator 1; **, perforator 2. **D** Intraoperative view after harvest of a 25 × 4 cm peroneal artery perforator flap without muscle dissection. **E** Immediate postoperative appearance after flap inset. **F** One-month postoperative photograph

A 70-year-old male presented with a 7 × 4 cm soft tissue defect on the lateral aspect of the left knee (Fig. 4A). Preoperative CTA (Fig. 4B) identified two perforators from the peroneal artery. Based on CTA data, perforator 1 was chosen for flap design (Fig. 4C). Intraoperative findings were consistent with the CTA-based plan, and a 25 × 4 cm peroneal artery perforator flap was harvested (Fig. 4D and E). The donor site was closed primarily without tension. The 1-month postoperative appearance is shown in Fig. 4F. Donor-site scar ulceration developed during follow-up and was treated with topical wound care, resolving without surgical revision. At the final 11-month clinical follow-up, the flap remained stable, with satisfactory contour and no additional complications.

#### Case 3

**Fig. 5:**
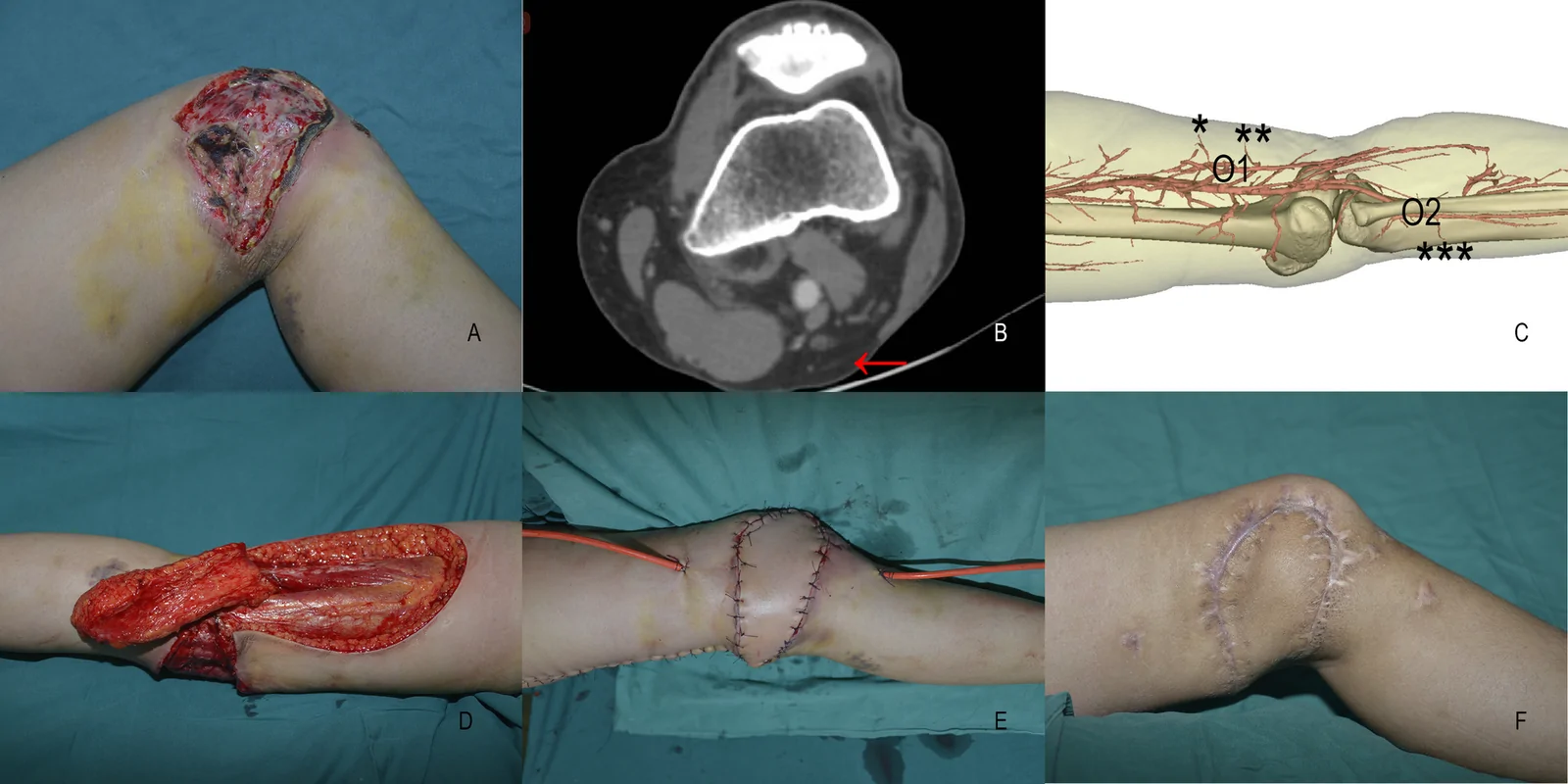
(**A**) Preoperative view of the soft tissue defect on the medial aspect of the left knee. **B** Preoperative CTA shows the location of perforator 2, indicated by the red arrow; this intermuscular perforator measured 2.1 cm in length. **C** Mimics 20.0 reconstruction showed two peri-popliteal perforators, one of which was interpreted as arising from the popliteal artery according to CTA-visible source-vessel continuity, and one anterior tibial artery perforator. O1: popliteal artery; O2: anterior tibial artery; *: perforator 1; **: perforator 2; ***: perforator 3. **D** An 18 × 7 cm peri-popliteal perforator flap was harvested without muscle separation. **E** Immediate postoperative appearance. **F** Photograph of the affected limb 1 year postoperatively

A 26-year-old female sustained a 12 × 7 cm medial knee defect (Fig. 5A) from a machine-related injury. Preoperative CTA (Fig. 5B) revealed three candidate perforators in the peri-popliteal region, with perforator 2 being the most suitable for flap design due to its proximity and favorable anatomy (Fig. 5C). This perforator was initially interpreted as arising from the popliteal artery according to CTA-visible source-vessel continuity and intraoperative findings; however, because small perforators in the peri-popliteal region may be anatomically close to branches of the sural arterial system, this flap is referred to as a peri-popliteal perforator flap in the present study. Intraoperative findings were consistent with the CTA-based plan, and an 18 × 7 cm peri-popliteal perforator flap was harvested (Fig. 5D). The flap was rotated to reconstruct the medial knee defect, with the immediate postoperative appearance shown (Fig. 5E). Partial distal necrosis involving less than 30% of the flap developed postoperatively and was managed with conservative wound care, healing without debridement, grafting, or further surgical intervention. At 1-year follow-up, the flap remained stable without additional complications (Fig. 5F).

## Discussion

In peri-knee soft tissue reconstruction, the principal challenge is not the lack of flap options but whether an appropriate perforator can be reliably identified and selected before incision. This issue is particularly relevant around the knee because local tissue tolerance is limited and functional demands are high; therefore, unexpected perforator anatomy, suboptimal perforator selection, or unfavorable pedicle geometry may complicate intraoperative decision-making and compromise reconstructive safety. Anatomical studies of the anterior knee region suggest multiple perforator-related angiosomes with substantial interindividual variation in their extent and distribution, consistent with the clinical observation that a “typical” perforator pattern cannot be assumed in every patient [12]. Although prior work has attempted to address this uncertainty using intraoperative algorithms or contingency-based decision-making, such strategies are inherently reactive: critical choices are often made only after surgical exposure, which may prolong operative time, increase dissection, and expand donor-site burden. These drawbacks are especially relevant in patients with active infection, compromised soft tissues, or limited physiological reserve [17].

The present survival profile and complication pattern should be interpreted as acceptable and broadly consistent with previous peri-knee perforator flap series rather than as evidence of superiority. Demirseren et al. reported survival of all 17 reverse-flow ALT perforator flaps, although two developed distal partial skin necrosis [3]. Hsieh et al. reported complete survival of five DGAP flaps for complex peripatellar defects [4]. Li et al. reported uneventful survival of 26 pedicled perforator flaps around the knee [25]. Differences in defect complexity, flap type, infection status, and endpoint definitions preclude direct comparison.

The core contribution of the present study is to describe how CTA-guided perforator assessment may shift key elements of reconstructive decision-making to the preoperative stage and translate anatomical variability into actionable choices. CTA delineates perforator origin, course, caliber, estimated pedicle length, and spatial relationship to the defect, enabling preoperative comparison among candidate perforators. Importantly, selection was anchored to practical criteria closely linked to the mechanics of peri-knee reconstruction, including proximity to the defect, adequate pedicle length and vessel caliber, flow direction when relevant to the planned design, and anticipated rotation angle. This approach may support formulation of a clearer reconstructive plan prior to incision; however, reductions in operative time, exploration burden, and dissection extent were not demonstrated in this study because no comparator cohort or standardized intraoperative efficiency metrics were available. In addition to identifying suitable perforators, CTA may also be useful for recognizing cases in which no reliable perforator is available or when the identified perforator is unsuitable for safe perforator-based flap design. In such cases, CTA may support timely selection of an alternative reconstructive strategy before incision. Furthermore, preoperative knowledge of pedicle length and rotational geometry helps tailor flap design to transverse or irregular defects. In such defects, insufficient pedicle length or excessive rotation increases the likelihood of pedicle kinking/torsion, impaired venous outflow, and marginal perfusion, thereby raising the risk of partial distal necrosis. In other words, in certain peri-knee scenarios, pedicle geometry may determine feasibility and safety margins more than the donor territory itself.

CTA should be regarded as a decision-support adjunct rather than a replacement for clinical assessment, handheld Doppler, or duplex/color Doppler ultrasound. Doppler-based methods remain useful because they are convenient, noninvasive, and provide real-time hemodynamic information, whereas CTA provides additional three-dimensional information on source vessels, deep perforator course, pedicle geometry, and spatial relationships to the defect [18]. In this series, the CTA-based workflow appeared feasible and qualitatively concordant with intraoperative findings, particularly in complex traumatic or infection-complicated wounds and in large-rotation or retrograde flap designs. In the retrograde ALT cases, no venous supercharging or additional venous procedure was routinely performed; however, the absence of clinically significant venous congestion should be interpreted cautiously because of the small sample size. Future prospective comparative studies are required to determine whether CTA-guided planning improves reproducibility, operative efficiency, or clinical outcomes.

## Limitations

Several limitations warrant consideration. First, this was a retrospective, single-center case series with a small sample size and substantial clinical heterogeneity, which may limit the generalizability of the findings. Second, no Doppler-only, non-CTA, or historical control group was included; therefore, this study cannot determine whether CTA-guided planning improves flap survival, reduces complication rates, shortens operative time, decreases intraoperative exploration, or reduces unplanned flap modification or conversion compared with other mapping strategies. Third, because the present cohort included only patients who underwent preoperative CTA, potential selection bias cannot be excluded. In particular, CTA may have been used preferentially in more complex defects, traumatic or infected wounds, or cases in which the surgeon anticipated greater uncertainty in perforator selection. Thus, the findings should be interpreted as reflecting the feasibility of a CTA-guided reconstructive decision-making cohort and the CTA–intraoperative concordance of CTA-selected perforator flaps, rather than the outcomes of all peri-knee reconstructions during the study period.

Fourth, objective intraoperative metrics were not systematically recorded. Operative time, duration of perforator exploration, extent of dissection, number of additional perforators explored, and time required for flap modification were not consistently available; therefore, the potential effect of CTA on operative efficiency could not be directly assessed. In addition, although available descriptive data were reported, complete patient-level source data were not consistently retrievable for all continuous variables. Therefore, median and standard deviation values for age, defect dimensions, flap dimensions, and follow-up duration could not be reliably calculated. To avoid introducing reconstructed or potentially inaccurate statistics, no imputation or retrospective estimation was performed. This limitation should be considered when comparing the present findings with other case series. Fifth, the 10-year inclusion period may have introduced a learning-curve effect. Improvements in surgeon experience, CTA interpretation, flap design, perioperative management, and microsurgical/perforator-flap techniques over time may have influenced outcomes independently of CTA-guided planning. Sixth, although CTA-mapped candidate perforator number and source vessels were recorded, standardized quantitative measurements of perforator diameter and intraoperative pedicle length were not available in all cases. Therefore, mean perforator diameter, mean pedicle length, interobserver reliability, and formal CTA–intraoperative quantitative correlation analyses could not be reported. Finally, CTA involves ionizing radiation and iodinated contrast exposure, which may limit its routine use, particularly in patients with renal insufficiency, contrast allergy, pregnancy, or when repeated imaging is undesirable. Accordingly, CTA should be considered a decision-support adjunct rather than a universal replacement for clinical assessment, handheld Doppler, or duplex/color Doppler ultrasound. Further prospective comparative studies with standardized intraoperative time metrics and clearly defined CTA indications are needed to determine whether CTA-guided planning improves operative efficiency or clinical outcomes.

## Conclusion

This retrospective Level IV case series demonstrates the feasibility of a structured CTA-based workflow for preoperative perforator assessment in individualized peri-knee soft tissue reconstruction. In this series, preoperative CTA delineation of perforator location, pedicle course, estimated pedicle length, vessel caliber, and the spatial relationship between the perforator and the defect showed qualitative concordance with intraoperative findings. The workflow may support individualized flap design, particularly in complex, irregular, traumatic, or infection-complicated peri-knee wounds. However, because no Doppler-only, non-CTA, or historical control group was included, the present study demonstrates feasibility and anatomical concordance rather than measurable clinical superiority. Further prospective comparative studies with standardized intraoperative metrics are required to determine whether CTA-guided planning improves operative efficiency, reduces intraoperative exploration or flap modification, or improves clinical outcomes.

## Data Availability

De-identified data, with all personal identifiers removed, may be made available from the corresponding author upon reasonable request and with approval from the institutional ethics committee, where applicable.

## Abbreviations

CTA: Computed tomography angiography
PACS: Picture Archiving and Communication System
ALT: Anterolateral thigh
STROBE: Strengthening the Reporting of Observational Studies in Epidemiology
DGF: Descending genicular artery perforator flap
RF: Random flap
SAF: Saphenous artery perforator flap
ALTF: Anterolateral thigh flap
SLGAF: Superior lateral genicular artery flap
PPF: Popliteal artery perforator flap
PAF: Peroneal artery perforator propeller flap
MSAP: Medial sural perforator propeller flap

## References

[1] Yamamoto T, Yamamoto N. A triple-component deep inferior epigastric artery perforator chimeric free flap for three-dimensional reconstruction of a complex knee defect complicated with patella osteomyelitis. Microsurgery. 2021;41(4):370–5. 33368468 10.1002/micr.30698

[2] Liu WC, et al. Reconstruction of massive knee defects with extensor mechanism deficiency through concurrent anterolateral thigh flap and autogenous hamstring tendon: A report of two cases. J Orthop Surg (Hong Kong). 2020;28(2):2309499020935994. 32730729 10.1177/2309499020935994

[3] Demirseren ME, et al. Clinical experience with a reverse-flow anterolateral thigh perforator flap for the reconstruction of soft-tissue defects of the knee and proximal lower leg. J Plast Reconstr Aesthet Surg. 2011;64(12):1613–20. 21784720 10.1016/j.bjps.2011.06.047

[4] Hsieh YH, et al. Reconstructing complex peripatellar defects using the descending genicular artery perforator flap. ANZ J Surg. 2023;93(7–8):1950–6. 37334914 10.1111/ans.18560

[5] Nguyen AT, et al. Lateral supragenicular pedicle perforator flap: clinical results and vascular anatomy. J Plast Reconstr Aesthet Surg. 2011;64(3):381–5. 20716494 10.1016/j.bjps.2010.05.029

[6] Tee R, et al. The medial sural artery perforator pedicled propeller flap for coverage of middle-third leg defects. J Plast Reconstr Aesthet Surg. 2019;72(12):1971–8. 31562028 10.1016/j.bjps.2019.08.006

[7] Lee SH, et al. An anatomical study of the saphenous nerve, artery, and artery perforators within the thigh using cadaveric dissection. Ann Plast Surg. 2011;67(4):413–5. 21407051 10.1097/SAP.0b013e31820bcd7b

[8] Poulet V, et al. Fibula free flap perforasomes: vascular anatomical study and clinical applications. Surg Radiol Anat. 2022;44(5):637–44. 35576016 10.1007/s00276-022-02953-4

[9] Zhao Y, et al. The clinical application and anatomical analysis of proximal peroneal artery perforator in free fibula flap based on CTA. BMC Oral Health. 2025;25(1):77. 39819466 10.1186/s12903-025-05433-4 PMC11737148

[10] Yu P, Selber J, Liu J. Reciprocal dominance of the anterolateral and anteromedial thigh flap perforator anatomy. Ann Plast Surg. 2013;70(6):714–6. 23364669 10.1097/SAP.0b013e318241446c

[11] Hirtler L, Lübbers A, Rath C. Vascular coverage of the anterior knee region - an anatomical study. J Anat. 2019;235(2):289–98. 31070789 10.1111/joa.13004 PMC6637446

[12] Saint-Cyr M, et al. The perforasome theory: vascular anatomy and clinical implications. Plast Reconstr Surg. 2009;124(5):1529–44. 20009839 10.1097/PRS.0b013e3181b98a6c

[13] Hallock GG. Direct and indirect perforator flaps: the history and the controversy. Plast Reconstr Surg. 2003;111(2):855–65. quiz 866. 12560714 10.1097/01.PRS.0000040462.38363.C7

[14] Lu TC, et al. Algorithmic approach to intraoperative conversion in free anterolateral thigh flap reconstruction. Plast Reconstr Surg. 2018;141(1):141–50.

[15] Du Q, et al. Distally Based Anterolateral Thigh Flap Algorithm for Unexpected Situations during Soft-Tissue Defect Reconstruction around the Knee. Plast Reconstr Surg. 2024;153(3):728–38. 37289943 10.1097/PRS.0000000000010814

[16] Du Q, et al. Improving the outcome of distally based anterolateral thigh flap reconstruction: New classification and surgical guidelines. J Plast Reconstr Aesthet Surg. 2023;87:229–37. 37918300 10.1016/j.bjps.2023.10.062

[17] Lu JC, et al. Algorithmic approach to anterolateral thigh flaps lacking suitable perforators in lower extremity reconstruction. Plast Reconstr Surg. 2015;135(5):1476–85. 25835248 10.1097/PRS.0000000000001168

[18] Feng S, et al. A Prospective Head-to-Head Comparison of Color Doppler Ultrasound and Computed Tomographic Angiography in the Preoperative Planning of Lower Extremity Perforator Flaps. Plast Reconstr Surg. 2016;137(1):335–47. 26710036 10.1097/PRS.0000000000001895

[19] Hallock GG. Doppler sonography and color duplex imaging for planning a perforator flap. Clin Plast Surg. 2003;30(3):347–57. 12916592 10.1016/s0094-1298(03)00036-1

[20] González Martínez J, et al. Preoperative Vascular Planning of Free Flaps: Comparative Study of Computed Tomographic Angiography, Color Doppler Ultrasonography, and Hand-Held Doppler. Plast Reconstr Surg. 2020;146(2):227–37. 32740566 10.1097/PRS.0000000000006966

[21] Masia J, et al. Multidetector-row computed tomography in the planning of abdominal perforator flaps. J Plast Reconstr Aesthet Surg. 2006;59(6):594–9. 16716952 10.1016/j.bjps.2005.10.024

[22] World Medical Association. Declaration of Helsinki. Ethical principles for medical research involving human subjects. Nurs Ethics. 2002;9(1):105–9. 16010903 10.1191/0969733002ne486xx

[23] Zhang YX, et al. The Economy in Autologous Tissue Transfer: Part 1. The Kiss Flap Technique. Plast Reconstr Surg. 2016;137(3):1018–30. 26910687 10.1097/01.prs.0000479971.99309.21

[24] Jeyaratnam S, Sebastin SJ, Das De SD. Revisiting the reconstructive ladder for soft tissue reconstruction in the lower extremity. Ann Transl Med. 2024;12(1):7. 38304896 10.21037/atm-23-1445 PMC10777235

[25] Li Z, Li P, Tan Q. Reconstruction of Soft Tissue Defects Around the Knee With Pedicled Perforator Flaps. Ann Plast Surg. 2018;81(4):462–7. 30124490 10.1097/SAP.0000000000001544

